# A Tissue-Engineered Tracheobronchial In Vitro Co-Culture Model for Determining Epithelial Toxicological and Inflammatory Responses

**DOI:** 10.3390/biomedicines9060631

**Published:** 2021-06-02

**Authors:** Luis Soriano, Tehreem Khalid, Fergal J. O’Brien, Cian O’Leary, Sally-Ann Cryan

**Affiliations:** 1 School of Pharmacy and Biomolecular Sciences, RCSI University of Medicine and Health Sciences, D02 YN77 Dublin, Ireland; luissoriano@rcsi.com (L.S.); TehreemKhalid@rcsi.ie (T.K.); cianoleary@rcsi.ie (C.O.); 2 Tissue Engineering Research Group, Department of Anatomy and Regenerative Medicine, RCSI University of Medicine and Health Sciences, D02 YN77 Dublin, Ireland; fjobrien@rcsi.ie; 3 SFI Centre for Research in Medical Devices (CÚRAM), RCSI University of Medicine and Health Sciences, D02 YN77 Dublin, Ireland; 4 SFI Advanced Materials and Bioengineering Research (AMBER) Centre, RCSI University of Medicine and Health Sciences and Trinity College Dublin, D02 YN77 Dublin, Ireland; 5 Trinity Centre for Biomedical Engineering, Trinity College Dublin, D02 PN40 Dublin, Ireland

**Keywords:** respiratory tissue engineering, collagen, toxicology, 3D in vitro models, co-culture, epithelium, lipopolysaccharide, bleomycin, air-liquid interface

## Abstract

Translation of novel inhalable therapies for respiratory diseases is hampered due to the lack of in vitro cell models that reflect the complexity of native tissue, resulting in many novel drugs and formulations failing to progress beyond preclinical assessments. The development of physiologically-representative tracheobronchial tissue analogues has the potential to improve the translation of new treatments by more accurately reflecting in vivo respiratory pharmacological and toxicological responses. Herein, advanced tissue-engineered collagen hyaluronic acid bilayered scaffolds (CHyA-B) previously developed within our group were used to evaluate bacterial and drug-induced toxicity and inflammation for the first time. Calu-3 bronchial epithelial cells and Wi38 lung fibroblasts were grown on either CHyA-B scaffolds (3D) or Transwell^®^ inserts (2D) under air liquid interface (ALI) conditions. Toxicological and inflammatory responses from epithelial monocultures and co-cultures grown in 2D or 3D were compared, using lipopolysaccharide (LPS) and bleomycin challenges to induce bacterial and drug responses in vitro. The 3D in vitro model exhibited significant epithelial barrier formation that was maintained upon introduction of co-culture conditions. Barrier integrity showed differential recovery in CHyA-B and Transwell^®^ epithelial cultures. Basolateral secretion of pro-inflammatory cytokines to bacterial challenge was found to be higher from cells grown in 3D compared to 2D. In addition, higher cytotoxicity and increased basolateral levels of cytokines were detected when epithelial cultures grown in 3D were challenged with bleomycin. CHyA-B scaffolds support the growth and differentiation of bronchial epithelial cells in a 3D co-culture model with different transepithelial resistance in comparison to the same co-cultures grown on Transwell^®^ inserts. Epithelial cultures in an extracellular matrix like environment show distinct responses in cytokine release and metabolic activity compared to 2D polarised models, which better mimic in vivo response to toxic and inflammatory stimuli offering an innovative in vitro platform for respiratory drug development.

## 1. Introduction

Respiratory diseases and related infections are one of the leading causes of mortality worldwide presenting a staggering economic and social burden, with estimates of four million premature annual deaths from chronic respiratory diseases [1]. This includes the five major respiratory conditions—acute respiratory infections, chronic obstructive pulmonary disease, asthma, tuberculosis, and lung-related cancers—which, along with significant morbidity and mortality, are believed to account for EUR 379.6 billion in healthcare costs annually within the European Union and USD 129 billion in the United States [2,3]. Even though the prevalence and deleterious effects of some respiratory illnesses can be avoided and/or mitigated by promoting public health care and primary prevention measures, novel treatment options are still urgently needed to decrease the burden of respiratory disease on health and society [4,5]; this has become increasingly apparent during the SARS-CoV-2 global pandemic. In order to develop new and better treatments, a deeper understanding of the complex pathophysiology of respiratory diseases and better tools with which to screen new therapeutics and formulations are required.

During the last decades, only a small number of novel therapies have been introduced as treatment options for respiratory diseases as it is estimated only 3% of new respiratory drugs reach final market and clinical approval [5]. Several factors hamper the progression of novel respiratory treatments into clinical applications including poor understanding of disease pathophysiology and the lack of proper in vitro models to assess drug efficacy and safety early in the development process. Moreover, it is crucial to determine efficient but safe concentrations of drugs delivered through the inhalation route that presents challenges due to the complex interplay of several pharmacokinetic processes taking place in the airways [6]. The complexity of the respiratory system provides many challenges to fully evaluate the impact of novel therapies and delivery systems in the intricate network of tissues involved in respiratory malfunction, which can be appreciated in the failure of many drugs candidates after in vivo testing [7]. Apart from ethical concerns regarding animal testing, it has been demonstrated that in vivo animal models might produce contradictory results and have poor value for translation into humans [8]. Moreover, the majority of pre-clinical in vitro models for drug assessment fail to recapitulate the complex physiology of the upper respiratory tract by not including relevant airway cell types as well as by not mimicking the three-dimensional (3D) environment in which respiratory tissues are located. The widespread use of two-dimensional (2D) cultures on plastic surfaces and filter cultures, which do not resemble in vivo microenvironments, are described as a key aspect in poor drug discovery outcomes [9]. The inclusion of 3D in vitro models could increase translation of experimental therapeutics by offering a more complex 3D environment that better mimics the extracellular matrix (ECM) surrounding respiratory tissues together with the inclusion of co-culture strategies. Therefore, there is an urgent need to develop more accurate and complex 3D models of the airway to be used as tools during drug discovery to augment clinical translation of novel therapies for respiratory medicine.

A key challenge in respiratory medicine is the development of advanced nanoparticulate therapies to ameliorate respiratory conditions and provide curative effects rather than only symptomatic relief. Robust models that enable the rapid testing of cellular responses to these new therapeutics, delivery systems and even pathogens are urgently needed. Recently, several tissue-engineered strategies have been developed consisting of biocompatible scaffolds with airway cells to better represent the native tissues of the airway [10,11]. The supporting scaffolds allow cells to deposit ECM components creating a relevant 3D environment, which in combination with relevant cell co-culture strategies e.g., fibroblasts and bronchial epithelial cells, allow for better understanding of epithelial responses of the human airway mucosa to foreign stimuli and/or inhaled formulations [12]. Our research group has recently developed an innovative collagen and hyaluronic acid bilayered (CHyA-B) scaffold that can support the formation of a submucosal tissue analogue of the conducting section of the lower respiratory tract. This model demonstrated an improved tracheobronchial phenotype over standard 2D monocultures and offers significant potential to study cellular responses in respiratory drug discovery and delivery [13].

Therefore, the major aim of this study was to harness this innovative bilayered scaffold to evaluate airway cell responses following bacterial and drug-mediated challenges. The scaffold was seeded with Calu-3 bronchial epithelial cells and Wi38 lung derived fibroblasts in co-culture under air–liquid interface (ALI) conditions, followed by analysis of biological response to several stresses and comparison to conventional 2D insert models. Overall, this study sought to determine if the tissue engineered model recapitulates physiologically-relevant toxicological and inflammatory responses to stimuli better than the current 2D standard.

## 2. Materials and Methods

### 2.1. Materials

Unless specified in the text, all materials and reagents were supplied by Sigma-Aldrich (Arklow, Ireland).

### 2.2. Collagen and Hyaluronic Acid Bilayered (CHyA-B) Scaffold Fabrication

Collagen and Hyaluronic Acid (CHyA) films were fabricated using a modification of a previously described method [13,14]. A suspension of 0.5% type I bovine tendon collagen (Collagen Solution, Glasgow, UK) and 0.004% hyaluronic acid sodium salt from *Streptococcus equi* in 0.5 M acetic acid (AcOH) (Fisher Scientific, Ballycoolin, Ireland) was blended using an Ultra Turrax T18 Overhead blender (IKA Works Inc., Wilmington, NC, USA). The CHyA slurry was degassed under a vacuum and was pipetted onto a 6 × 6 cm^2^ polytetrafluoroethylene plate to produce a thin and transparent CHyA copolymer film. CHyA-B scaffolds were manufacture by freeze-drying CHyA films combined with CHyA slurry using a customised metal mould as previously described [13,15]. CHyA films were rehydrated in AcOH and the slurry-film combination was freeze dried using a customised anneal cycle that initially froze the scaffolds to −20 °C, before increasing shelf temperature to −10 °C, at which it was held at for 24 h prior to sublimation [16]. After freeze-drying, CHyA-B scaffolds were crosslinked using a dehydrothermal (DHT) process at 105 °C during 24 h at 50 mTorr (VacuCell 22, MMM, Planegg, Germany) [17]. CHyA-B scaffolds were cut into discs with a diameter of 15.6 mm^2^ and chemically crosslinked using a 5:2 molar ratio of 1-ethyl-3-(3-dimethylaminopropyl) carbodiimide (EDAC) and N-Hydroxysuccinimide (NHS) [16,18].

### 2.3. Cell Culture

#### 2.3.1. Cell Selection and Culture Media

The Calu-3 bronchial epithelium cell line (ATCC, Middlesex, UK) was cultured in a 1:1 mixture of Dulbecco’s modified Eagle’s medium and Ham’s F12 medium supplemented with 10% foetal bovine serum (Biosera, Ringmer, UK), 2 mM l-glutamine, 14 mM sodium bicarbonate, and 100 U/mL penicillin/streptomycin; this was referred to as Calu-3 medium. Cells were used between passages 20–38. The Wi38 human embryonic lung fibroblast cell line (ATCC) was cultured in Eagle’s minimal essential medium supplemented with 10% foetal bovine serum, 2 mM l-glutamine, 26 mM sodium bicarbonate, 100 U/mL penicillin/streptomycin, and 1 mM sodium pyruvate; this was referred to as Wi38 medium. Cells were used between passages 18–24. Co-culture medium used was a 1:1 mixture of Calu-3:Wi38 medium. Unless otherwise stated, all cell culture and incubation steps were performed at 37 °C and 5% CO_2_ in a humidified atmosphere.

#### 2.3.2. Non-Polarised Epithelial Culture

Calu-3 cells were seeded into a 12 well plate with 5 × 10^5^ cells/cm^2^ and grown for two days before exposure to Lipopolysaccharide (LPS). Each well was pre-warmed with 500 μL of Calu-3 medium for 30 min at 37 °C and then seeded using 1 mL of medium containing 5 × 10^5^ cells.

#### 2.3.3. Polarised Epithelial Culture on Transwell^®^ Inserts

**Figure 1:**
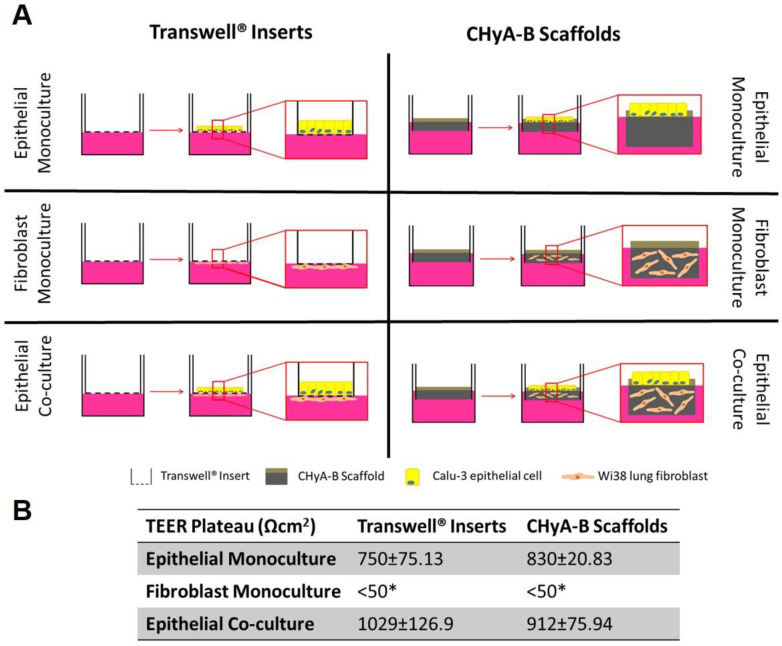
Evaluation of epithelial barrier integrity in air liquid interface (ALI) culture models used for exposure studies. (**A**) Calu-3 epithelial monocultures, Wi38 fibroblast monocultures, and Calu-3 Wi38 epithelial co-cultures on Transwell^®^ inserts and CHyA-B scaffolds grown under ALI conditions for 14 days. (**B**) Average TEER values of Calu-3 epithelial cell barriers following plateau of electrical resistance (day 10, 12 and 14). (* TEER measurements for fibroblast monocultures on Transwell^®^ inserts and CHyA-B scaffolds were performed as *n* = 2) Results displayed as mean ± SEM (*n* = 7).

For epithelial monocultures, Calu-3 epithelial cells were seeded onto the apical side of 12 mm Transwell^®^ inserts (VWR, Dublin, Ireland) as shown in Figure 1A. Briefly, 1 mL of co-culture medium was transferred onto the basolateral compartment and 400 μL into the apical compartment. The apical compartment of the Transwell^®^ insert was then seeded with 5.65 × 10^5^ Calu-3 cells in 100 μL of Calu-3 medium and incubated for three days, which equals to 5 × 10^5^ cell/cm^2^. At day three of culture, medium from the apical compartment was removed to introduce ALI conditions. Cells on Transwell^®^ inserts were fed every 2–3 days with 500 μL of co-culture medium in the basolateral compartment. For co-culture experiments, Wi38 fibroblasts were initially seeded followed by Calu-3 addition as previously described with 3 × 10^4^ cells/cm^2^ [13]. Transwell^®^ inserts were initially inverted and seeded with 3.36 × 10^4^ cells in 50 μL of Wi38 medium and incubated for 2 h to encourage cell attachment to the membrane. Thereafter, seeded inserts were returned to wells containing 1 mL of Wi38 medium and grown for 2 days. Two days after fibroblast seeding, inserts were seeded with Calu-3 cells on the apical side as previously described. For some experiments, Transwell^®^ inserts seeded only with Wi38 fibroblast on the basolateral side were used and referred as fibroblast monoculture on Transwell^®^ inserts.

#### 2.3.4. Polarised Epithelial Culture on CHyA-B Scaffolds

For epithelial monocultures, Calu-3 epithelial cells were seeded onto the apical side of 12 mm Snapwell^®^ cell insert (VWR) as shown in Figure 1A, as previously described [13]. After chemical crosslinking, CHyA-B scaffolds of 15.6 mm-diameter and 1 mm in height were pinned into the frame of a Snapwell^®^ cell insert and preconditioned using 3 mL of co-culture medium in the basolateral compartment and 400 μL in the apical compartment. The apical side of the scaffold was seeded using 5.65 × 10^5^ Calu-3 cells in 100 μL of Calu-3 medium and incubated for three days, which equals to 5 × 10^5^ cell/cm^2^. At day three of culture, medium from the apical compartment was removed to introduce ALI and samples were fed every 2–3 days with 2 mL of co-culture medium in the basolateral compartment. For co-culture experiments, the porous layer of each scaffold was pre-warmed with 3 mL of co-culture medium and seeded with 6 × 10^6^ Wi38 cells in 50 μL of co-culture medium. Samples were incubated for 2 h to encourage cell attachment and supplemented with 3 mL of fresh medium. After 2 days of cell acclimatisation to the scaffold, they were pinned into the frame of a Snapwell^®^ cell insert and seeded with Calu-3 cells as previously outlined.

### 2.4. Evaluation of Epithelial Barrier Integrity

#### Transepithelial Electrical Resistance (TEER) Measurements

Transepithelial electrical resistance (TEER) values from epithelial monocultures and co-cultures on Transwell^®^ inserts and CHyA-B scaffolds were quantified throughout the cell culture period to monitor epithelial barrier integrity. Fresh medium was added to the apical and basolateral compartments and TEER was measured using an EVOM voltohmmeter (World Precision Instruments, Hitchin, UK), STX-2 chopstick electrodes (World Precision Instruments) for Transwell^®^ inserts and STX-3 chopstick electrodes (World Precision Instruments) for CHyA-B scaffolds. Transwell^®^ inserts were filled with 500 μL of fresh co-culture medium in the apical and 1 mL in the basolateral compartment, while 500 μL were added to the apical side of CHyA-B scaffolds and 2 mL to the basolateral compartment. Electrical resistance on cultures was measured on day 3, 5, 8, 10, 12 and 14 of culture. TEER values were calculated by subtracting the resistance of a cell-free scaffold or insert and corrected to the surface area (1.12 cm^2^) available for cell growth. TEER measurements for fibroblast monocultures on Transwell^®^ inserts and CHyA-B scaffolds were performed as *n* = 2 due to the inability of fibroblast to form tight junctions and, therefore, produce relevant TEER values [19].

### 2.5. Bacterial and Drug Challenge Studies

#### 2.5.1. Reagent Selection and Cell Exposure Optimisation

Cell-seeded samples were exposed to a selection of drugs and toxicants in order to evaluate the toxicological and inflammatory responses in both CHyA-B and Transwell^®^ cultures. LPS (derived from *Escherichia coli* and *Pseudomonas aeruginosa)* and bleomycin were selected to analyse responses to bacterial and drug toxicity, respectively. Bleomycin is an antibiotic used for the treatment of cancer with a significant side effect profile including fatal pulmonary toxicity and is a standard reagent used for preclinical respiratory toxicology [20]. Exposure conditions were optimise to provide an adequate toxicological and inflammatory challenge to epithelial cultures.

Non-polarised Calu-3 cells were exposed to LPS at a range of concentrations (10, 1 and 0.1 μg/mL) for 24 h. LPS was diluted to the desired concentration in 500 μL of 1% (serum starvation) and 10% serum (non-serum starvation) Calu-3 medium and added to non-polarised Calu-3 cells. Interleukin-8 (IL-8) secretion was detected using ELISA MAX™ Deluxe Set Human IL-8 (BioLegend, London, UK) per manufacturer instructions to evaluate the inflammatory response of Calu-3 cells following LPS exposure. Once an optimal LPS dose and exposure conditions were selected, polarised Calu-3 cells in Transwell^®^ inserts under ALI conditions were exposed to 10 μg/mL of LPS derived from *P. aeruginosa* for 24 h and 48 h in the apical compartment using 1% serum Calu-3 medium and IL-8 release was evaluated.

Polarised Calu-3 cells on Transwell^®^ inserts under ALI were exposed to bleomycin sulphate from *Streptomyces verticillus* (Sigma) at a range of concentrations (100, 10 and 1 μg/mL) for 24 h in 500 μL of Calu-3 medium. Cell metabolic activity, IL-8 secretion and TEER recovery were measured at day 15, 17, 19, 21 and 23 following exposure. Cell metabolic activity was quantified using alamarBlue™ Cell Viability Reagent (Biosciences, Dublin, Ireland) per manufacturer instructions. TEER recovery values were defined using the following equation:

$$\%\;\Delta \mathrm{TEER}=\frac{\frac{\mathrm{TEER}\;\left(f,t\right)}{\mathrm{TEER}\;\left(i,t\right)}}{\frac{\mathrm{TEER}\;\left(f,u\right)}{\mathrm{TEER}\;\left(i,u\right)}}\times 100$$

where TEER (*f*,*t*) is the final TEER value measured of treated samples, TEER (*i*,*t*) is the mean TEER value measured before exposure on day 10, 12 and 14 of treated samples, TEER (*f*,*u*) is the final TEER value measured of untreated samples and TEER (*i*,*u*) is the mean TEER value measured before exposure on day 10, 12 and 14 of untreated samples.

#### 2.5.2. Cell Toxicity Assays

Following relevant apical exposure period to 10 μg/mL LPS and 100 μg/mL bleomycin in 1% serum Calu-3 medium, cell toxicity following exposure was assessed using alamarBlue™ Cell Viability Reagent and Invitrogen™ CyQUANT™ LDH Cytotoxicity Assay (Fisher Scientific) to analyse the effect of the culture system on cell responses. Firstly, apical and basolateral samples were collected and lactate dehydrogenase (LDH) release was assessed. Secondly, samples were incubated with alamarBlue™ to evaluate cell metabolic activity. All kits were used according to the manufacturer’s instructions.

#### 2.5.3. Secretion of Pro-Inflammatory Cytokines

Secretion of interleukin-8 (IL-8), interleukin-6 (IL-6) and tumour necrosis factor alpha (TNF-α) was assessed by enzyme-linked immunosorbent assay (ELISA). Apical and basolateral samples were collected and centrifuged at 1200 rpm for 5 min before storage at −20 °C prior to assay evaluation. Samples were assayed for IL-8, IL-6 and TNF-α using the ELISA MAX™ Deluxe Set Human IL-8, IL-6 and TNF-α (BioLegend).

### 2.6. Data Analysis

Quantitative data obtained was analysed using Microsoft Excel and GraphPad Prism 7.0 Software. In cases of analysis between two groups, statistical difference was assessed by two-tailed Student *t*-test. For multiple groups, statistical difference between groups was assessed by 2-way ANOVA. For analysis of the TEER measurements, repeated measures were performed as part of the 2-way ANOVA.

## 3. Results

### 3.1. Evaluation of Epithelial Barrier Integrity

Formation of a functional epithelial barrier was evaluated by measuring TEER from epithelial monocultures and co-cultures on Transwell^®^ inserts and CHyA-B scaffold (Figure S1). Both epithelial monocultures and co-cultures were observed to produce TEER values higher than 600 Ω cm^2^ which characterises a functional epithelial barrier, with fibroblast monocultures showing negligible TEER < 50 Ω cm^2^ (Figure 1B and Figure S1B). The inclusion of fibroblast in Transwell^®^ inserts significantly increased the average TEER from 750.2 ± 75.13 Ω cm^2^ in epithelial monocultures to 1029 ± 126.9 Ω cm^2^ in epithelial co-cultures. Fibroblast addition to CHyA-B scaffolds led to an increase in TEER values from 830 ± 20.83 Ω cm^2^ in epithelial monocultures to 912 ± 75.94 Ω cm^2^ in epithelial co-cultures but this was not statistically significant (Figure 1B and Figure S1B). A polarised and functional epithelial barrier was formed in both Transwell^®^ inserts and CHyA-B scaffolds with similar values to those previously reported and the presence of fibroblasts in co-cultures appeared to improve epithelial barrier integrity [13].

### 3.2. Bacterial-Mediated Toxicity and Inflammation

#### 3.2.1. Optimisation of Bacterial Exposure Conditions

Non-polarised Calu-3 epithelial cells grown on culture dishes were exposed to LPS derived from *E. coli* and *P. aeruginosa* using three different concentrations: 10, 1, and 0.1 μg/mL for 24 h. Epithelial response to bacterial challenge was evaluated by assessing the release of IL-8, a key pro-inflammatory respiratory cytokine [21,22]. LPS derived from *E. coli* and *P. aeruginosa* lead to a significant increase in IL-8 secretion in both apical and basolateral compartments when non-polarised epithelial Calu-3 cultures were exposed to 1 and 10 μg/mL of LPS. However, *P. aeruginosa*-derived LPS was found to induce a more consistent dose response in comparison to *E. coli* and, therefore, was selected for further bacterial challenge optimisation (Figure S2A). Serum-starvation supported a more defined dose-response following LPS exposure in comparison to non-serum starvation conditions (Figure S2B). In order to determine optimal exposure time, polarised Calu-3 epithelial cells seeded on Transwell^®^ inserts were challenged with 10 μg/mL of LPS (*P. aeruginosa)* for 24 or 48 h from the apical compartment (Figure S2C). An exposure time of 24 h under serum starvation conditions led to a statistically significant increase in IL-8 secretion. Therefore, exposure of cells to 10 μg/mL LPS *(P. aeruginosa*) for 24 h under serum starvation conditions were used as standard exposure conditions for further bacterial challenge studies.

#### 3.2.2. Cell Toxicity after LPS Exposure

**Figure 2:**
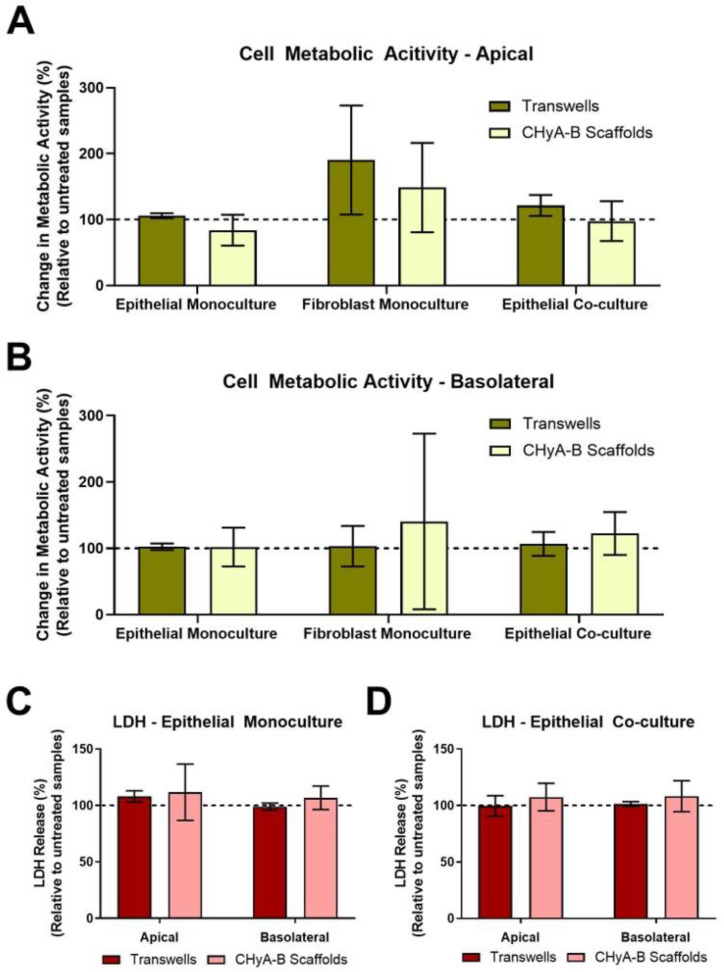
Cell viability of epithelial monocultures and co-cultures grown on Transwell^®^ inserts and CHyA-B scaffolds following bacterial challenge with 10 μg/mL of *P. aeruginosa* derived LPS for 24 h. Apical (**A**) and basolateral (**B**) cell metabolic activity of Calu-3 epithelial monocultures, Wi38 fibroblast monocultures and Calu-3 Wi38 epithelial co-cultures on Transwell^®^ inserts and CHyA-B scaffolds. Apical and basolateral LDH release from Calu-3 epithelial monocultures (**C**) and Calu-3 Wi38 epithelial co-cultures (**D**) on Transwell^®^ inserts and CHyA-B scaffolds following LPS exposure. Changes in metabolic activity and LDH release are relative to untreated samples for each culture condition. Results displayed as mean ± SEM (*n* ≥ 3).

The cell metabolic activity and LDH release of epithelial monocultures, fibroblast monocultures and epithelial co-cultures when grown on Transwell^®^ inserts and CHyA-B scaffolds was evaluated 24 h after LPS exposure (Figure 2*)*. Relative LDH release and cell metabolic activity from cultures grown on Transwell^®^ inserts and CHyA-B scaffolds was assessed in the apical and basolateral compartments. As shown, cultures remained viable and metabolically active on both Transwell^®^ inserts and CHyA-B scaffolds following LPS treatment relative to untreated cultures (Figure 2A,B). Equally, relative levels of LDH in treated cultures compared to untreated cultures grown on Transwell^®^ inserts and CHyA-B scaffolds did not show any significant difference following LPS exposure (Figure 2C,D). TEER measurements following LPS exposure were performed to assess barrier integrity as uncontrolled cell death can cause TEER reduction [23]. Albeit an initial drop in barrier integrity compared to untreated cultures was detected following exposure, no significant differences were observed between epithelial cultures grown on Transwell^®^ inserts and CHyA-B scaffolds with a sufficient recovery of barrier properties achieved after LPS exposure at day 23 (Figure S4).

#### 3.2.3. Inflammatory Response to LPS

**Figure 3:**
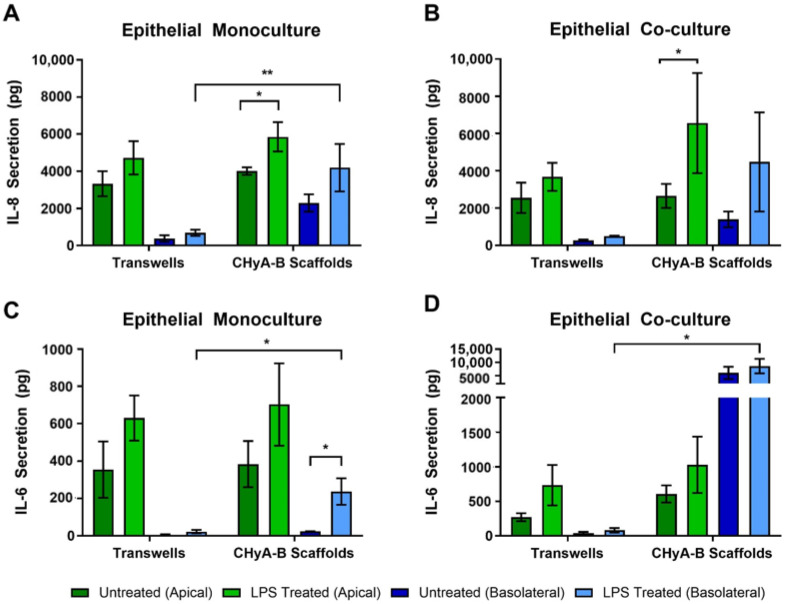
Pro-inflammatory cytokine release profile from epithelial cultures following LPS exposure. Apical and basolateral IL-8 release from Calu-3 monocultures (**A**) and Calu-3 Wi38 co-cultures (**B**) on Transwell^®^ inserts and CHyA-B scaffolds following LPS exposure with 10 μg/mL of *P. aeruginosa* derived LPS for 24 h. Apical and basolateral IL-6 release from Calu-3 epithelial monocultures (**C**) and Calu-3 Wi38 co-cultures (**D**) on Transwell^®^ inserts and CHyA-B scaffolds following LPS exposure. Results displayed as mean ± SEM (*n* = 3; * *p* < 0.05, ** *p* < 0.01).

To evaluate the inflammatory effect of LPS in epithelial monocultures and co-cultures in Transwell^®^ inserts and CHyA-B scaffolds, the release into the apical and basolateral compartments of the pro-inflammatory cytokines IL-8 and IL-6 was measured 24 h before exposure (Figure 3). All cultures grown on Transwell^®^ inserts and CHyA-B showed an increase in IL-8 secretion following LPS challenge in comparison to the relevant untreated condition (Figure 3A,B). The apical secretion of IL-8 in epithelial monocultures was similar for Transwell^®^ inserts and CHyA-B cultures. However, addition of co-culture conditions led to a greater increase in IL-8 secretion from CHyA-B compared to Transwell^®^ cultures relative to untreated ones. Basolateral secretion of IL-8 from epithelial monocultures was significantly different between CHyA-B (2293 ± 934 pg in untreated cultures, 4191 ± 2555 pg in LPS-treated cultures) and Transwell^®^ cultures (378 ± 349 pg in untreated cultures, 688 ± 335 pg in LPS-treated cultures) (Figure 3A). However, this differential basolateral profile in IL-8 secretion was not observed in Calu-3 Wi38 epithelial co-cultures (Figure 3B).

As for IL-8, release of IL-6 from Transwell^®^ and CHyA-B epithelial monocultures was similar (Figure 3C). However, the addition of Wi38 fibroblasts led to an increased IL-6 secretion from CHyA-B compared to Transwell^®^ cultures (Figure 3D). Low basolateral levels of IL-6 were detected in untreated Transwell^®^ and CHyA-B cultures. A significant increase in IL-6 secretion basolaterally from CHyA-B cultures following LPS exposure was detected which could not be observed in Transwell^®^ cultures (Figure 3C). The addition of co-culture conditions led to a significantly higher basolateral increase of IL-6 release from CHyA-B cultures both untreated and LPS treated (6034 ± 4036 pg in untreated cultures, 8615 ± 4712 pg in LPS-treated cultures) in comparison to Transwell^®^ cultures (37 ± 32 pg in untreated cultures, 81 ± 57 pg in LPS-treated cultures) (Figure 3D). TNF-α secretion from all cultures was not detectable (data not shown).

### 3.3. Drug-Mediated Toxicity and Inflammation

#### 3.3.1. Optimisation of Drug-Mediated Exposure Conditions

Polarised Calu-3 epithelial cells grown on Transwell^®^ inserts under ALI conditions were exposed to 1, 10 and 100 µg/mL of bleomycin during 24 h from the apical compartment, an anticancer chemotherapy drug widely used in airway epithelial research to create induced lung injury models [24,25]. Cell metabolic activity, IL-8 secretion and TEER measurements from polarised epithelial cells were assessed at day 15, 17, 19, 21 and 23 following exposure to evaluate cell toxicity, airway inflammation and barrier integrity (Figure S3). Apical and basolateral cell metabolic activity from cultures treated with 10 and 100 µg/mL of bleomycin were significantly reduced relative to untreated cultures at all time points tested. The greatest decrease in cell metabolic activity was detected on day 23 following exposure to 100 µg/mL bleomycin with a 43.86 ± 0.7% apical and 37.89 ± 2.1% basolateral reduction compared to untreated cultures (Figure S3A,B). IL-8 secretion from polarised cultures was significantly increased relative to untreated samples at day 21 and 23 after 100 µg/mL bleomycin exposure (Figure S3C) and a significant decreased in TEER was observed at day 23 following 100 µg/mL bleomycin exposure (Figure S3D). Consequently, exposure to 100 µg/mL bleomycin for 24 h was selected as the standard exposure conditions for further drug-mediated toxicity challenge studies.

#### 3.3.2. Cell Toxicity to Bleomycin

**Figure 4:**
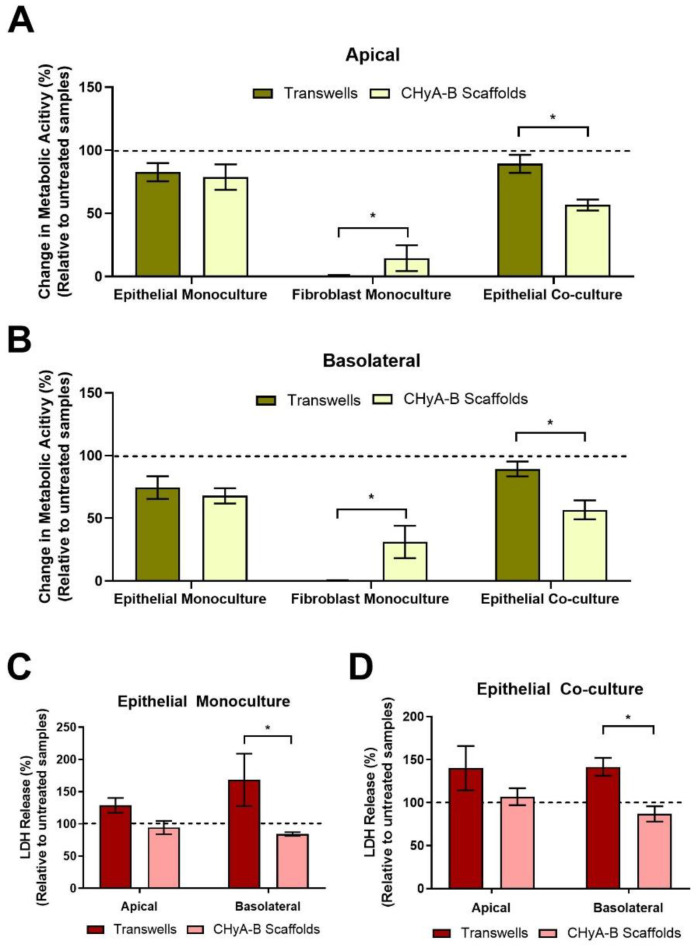
Cell viability on Transwell^®^ inserts and CHyA-B scaffolds following drug-mediated challenge. Apical (**A**) and basolateral (**B**) cell metabolic activity of Calu-3 epithelial monocultures, Wi38 fibroblast monocultures, and Calu-3 Wi38 epithelial co-cultures on Transwell^®^ inserts and CHyA-B scaffolds following exposure to 100 μg/mL of bleomycin for 24 h and measured at day 23 in culture. Apical and basolateral LDH release from Calu-3 epithelial monocultures (**C**) and Calu-3 Wi38 epithelial co-cultures (**D**) on Transwell^®^ Inserts and CHyA-B scaffolds following bleomycin exposure. Changes in metabolic activity and LDH release are relative to untreated samples for each culture condition. Results displayed as mean ± SEM (*n* ≥ 3; * *p* < 0.05).

Differential epithelial toxicological responses arising from exposure to bleomycin between culture conditions were compared by assessing the metabolic cell activity and LDH release of Transwell^®^ and CHyA-B epithelial monocultures and co-cultures (Figure 4). Similar decreases in metabolic activity in the apical and basolateral compartment were seen in epithelial Transwell^®^ and CHyA-B monocultures following drug-mediated challenge. In contrast, a significant drop in metabolic activity was seen in co-cultures grown on CHyA-B though not in Transwell^®^ cultures (Figure 4A,B). In addition, significant differences were seen between basolateral LDH release from epithelial monocultures and co-cultures grown on CHyA-B scaffolds compared to Transwell^®^ inserts post bleomycin exposure (Figure 4C,D). Transwell^®^ epithelial monocultures showed increased LDH release (168.47 ± 70.4%) relative to untreated samples in comparison to a decrease (84.44 ± 4.86%) for CHyA-B epithelial monocultures (Figure 4C). This significant difference in cellular response to the bleomycin challenge between cultures was also seen for co-cultures grown on different supports (Figure 4D).

**Figure 5:**
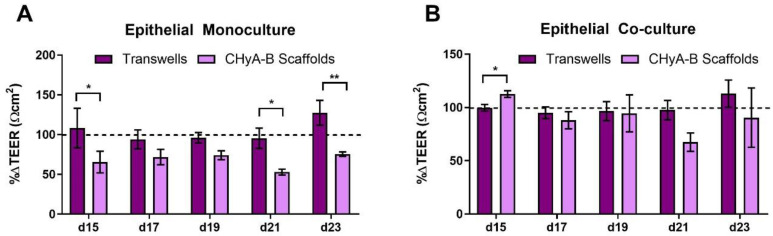
TEER measurements following drug-mediated challenge. TEER recovery of Calu-3 epithelial monocultures (**A**) and Calu3 Wi38 epithelial co-cultures (**B**) on Transwell^®^ inserts and CHyA-B scaffolds following challenge with 100 μg/mL of bleomycin for 24 h measured at day 15, 17, 19, 21 and 23 in culture. TEER recovery values normalised to the recovery of untreated samples for each culture condition. Results displayed as mean ± SEM (*n* = 3; * *p* < 0.05, ** *p* < 0.01).

An initial reduction in TEER was seen in epithelial monocultures on CHyA-B scaffolds immediately after exposure. Furthermore, TEER measurements showed a decreased recovery in epithelial barrier integrity on CHyA-B scaffolds in comparison to Transwell^®^ inserts at day 21 and 23 after exposure relative to untreated cultures (Figure 5A). Interestingly, Transwell^®^ and CHyA-B co-cultures were able to recover showing no significant decrease in TEER in comparison to untreated co-cultures (Figure 5B).

#### 3.3.3. Inflammatory Response

**Figure 6:**
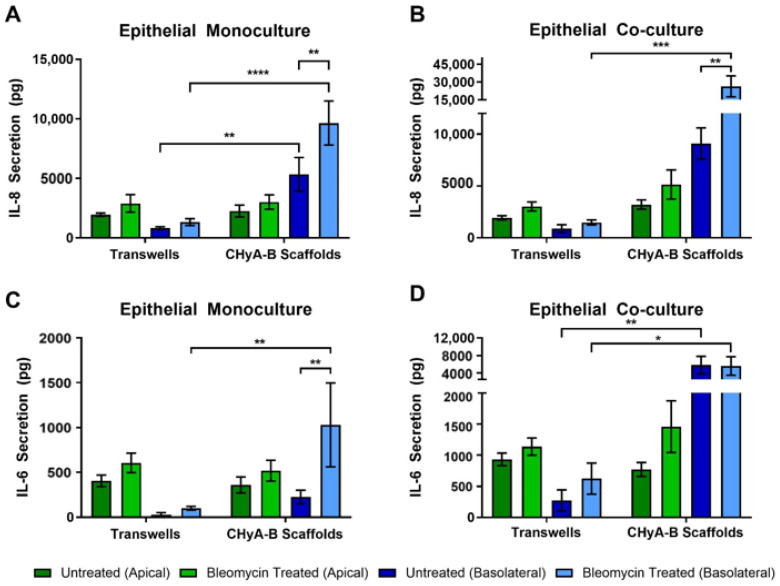
Pro-inflammatory cytokine release profile from epithelial cultures following drug-mediated challenge. Apical and basolateral IL-8 release from Calu-3 epithelial monocultures (**A**) and Calu-3 Wi38 co-cultures (**B**) on Transwell^®^ inserts and CHyA-B scaffolds following challenge with 100 μg/mL of bleomycin for 24 h measured at day 23 in culture. Apical and basolateral IL-6 release from Calu-3 epithelial monocultures (**C**) and Calu-3 Wi38 co-cultures (**D**) on Transwell^®^ inserts and CHyA-B scaffolds following bleomycin exposure. Results displayed as mean ± SEM (*n* = 3; * *p* < 0.05, ** *p* < 0.01, *** *p* < 0.001, **** *p* < 0.0001).

To further asses the effect of bleomycin on epithelial cultures, the production of pro-inflammatory cytokines in response to drug-mediated challenge was measured after exposure, at day 23 (Figure 6). IL-8 secretion was found to be increased following bleomycin treatment in all Transwell^®^ and CHyA-B cultures (Figure 6A,B). However, basolateral levels of IL-8 were increased from CHyA-B epithelial monocultures in comparison to Transwell^®^ monocultures in both untreated and bleomycin-treated cultures (Figure 6B). This relative increase in IL-8 expression when cells were cultured on the CHyA-B scaffolds was also seen for epithelial co-cultures (Figure 6B). Most notably, only cultures grown on CHyA-B scaffolds were able to detect a statistically significant increase in IL-8 expression post-bleomycin treatment ***(***Figure 6A,B).

Bleomycin increased the secretion of IL-6 after exposure in comparison to the relative untreated controls, although only significant for CHyA-B monocultures (Figure 6C,D). Apical secretion showed no significant differences between Transwell^®^ and CHyA-B epithelial monocultures, but inclusion of Wi38 fibroblasts led to a doubling of IL-6 secretion in Transwell^®^ and CHyA-B co-cultures (Figure 6C,D). Basolateral secretion of IL-6 from Transwell^®^ epithelial monocultures remained low with a significantly higher secretion observed in CHyA-B scaffolds (Figure 6C). Furthermore, basolateral IL-6 levels from CHyA-B epithelial co-cultures were significantly higher in comparison to Transwell^®^ epithelial co-cultures, but no significant increase in IL-6 was seen following bleomycin exposure (Figure 6C). TNF-α secretion from all cultures was not detectable (data not shown).

## 4. Discussion

The use of polarised epithelial models of the tracheobronchial region are well-established and support a polarised airway epithelium to mimic in vivo physiological processes and inhalation studies dynamics [26]. However, upgrading of 2D models of the airway is needed to fully reflect epithelial responses in a way closer to in vivo behaviour in order to facilitate drug development and better understand cellular responses in the airway. The use of conventional 2D models, such as cell culture inserts, e.g., Transwell^®^, are limited by the lack of a ECM-like 3D environment that better recapitulates epithelial responses to biochemical and biophysical stimuli [27]. Moreover, such conventional airway in vitro models do not fully recapitulate the multicellular in vivo environment in the lungs as single cell type cultures are often used. As with other human tissues, the lining of the lower respiratory tract contains a wide variety of cell types that can be better recapitulated using co-culture models of the airway. In this way, complex cell to cell communication and crosstalk between cell types can better represent epithelial responses against toxicological and inflammatory process and match more closely those of a complex in vivo system [7,28,29]. Therefore, the purpose of this study was to evaluate a previously developed tissue engineered bilayered scaffold of the tracheobronchial region as an in-vitro model to more closely mimic the epithelial responses against bacterial and drug-mediated toxicological and inflammatory challenges [13].

Polarised Calu-3 epithelial cells grown on Transwell^®^ inserts have become a standard model to study airway cell interactions to inhaled pathogens as well as other stimuli causing inflammation and epithelial cell toxicity [30]. LPS has been widely used as a positive control to generate inflamed models of the tracheobronchial region, but there is a lack of standard exposure conditions across the literature. This hampers the development of robust inflammatory in vitro models of the respiratory tract and accurate comparison between them [31]. Herein, a systematic optimisation of challenge conditions was undertaken to study the effect of LPS on the secretion of the pro-inflammatory cytokine IL-8 using different conditions including different concentrations, serum concentrations in media and exposure times in non-polarised and polarised Calu-3 epithelial cells on Transwell^®^ inserts (Figure S2). Our findings showed that a concentration of 10 µg/mL of *P. aeruginosa*-derived LPS in serum starvation conditions for 24 h was suitable for the creation of a robust bacterial challenge in a polarised epithelial airway model.

Drug-mediated toxicity and inflammation of the airway epithelium are commonly studied using bleomycin lung injury animal models, a potent anticancer drug that causes pulmonary fibrosis due to changes in fibroblast phenotypes [32]. These are associated with ethical concerns and poor translation due to anatomical and physiological differences between humans and animal models [33]. Therefore, new in vitro models for drug-mediated injury on human tissues could provide much needed tool for development. To the best of our knowledge, this is the first report of the effect of bleomycin exposure on polarised Calu-3 epithelial cells. Again, the conditions of exposure were systemically optimised. Decreased metabolic activity, increased IL-8 secretion and poor TEER recovery following 100 µg/mL bleomycin exposure on Calu-3 cells grown on Transwell^®^ inserts was determined to provide a suitable challenge condition (Figure S3). These cellular responses exposure in vitro correlates with bleomycin-induced lung injury models, in which inflammation and increased cell apoptosis are seen in the first 7 days after intratracheal administration [34]. Inflammation observed in this 2D polarised model also resembles the increased secretion of cytokine from murine models with high release of pro-inflammatory cytokines 9 days after exposure [35].

ALI culture conditions for epithelial cells have been shown to support more realistic cell to cell interactions and cell responses towards potential lung toxicants, which finally enable better testing of drug formulations and assessment of inhaled toxicity [36,37,38]. For example, polarised models allow for maintenance of a mucus layer over the epithelial monolayer, a key featured involved in the deposition and uptake of inhaled nanoparticles [39]. Overall, these models better predict cell toxicity, inflammation and barrier integrity–the main outputs herein examined after epithelial exposure [29,40]. These polarised models can be further improved by culturing epithelial cells in a 3D environment that resembles the ECM present in physiological conditions allowing for cell to ECM interaction that is not completely modelled in 2D models [41]. The presence of an ECM-like environment better recapitulates tissue architecture as well as mechanical and biomechanical cues. Moreover, it has been widely reported that cell to ECM interactions establish a 3D communication network which allows for tissue homeostasis maintenance modulating cell proliferation, migration, and apoptosis [42,43]. Therefore, it is crucial to include 3D environments in in vitro airway models. The use of co-cultures or even tri-cultures, e.g., lung fibroblasts, bronchial fibroblast, macrophages and mesenchymal stem cells into airway in vitro models has been explored over the last decade to better understand crosstalk between epithelial cells and underlying cell populations [12,29,44,45,46,47]. For instance, inclusion of fibroblasts has been reported to increase proliferation and differentiation of co-cultured epithelial cells while inclusion of a variety of immune cells allows for better interpretation of crosstalk at the epithelial barrier [48,49,50]. Inclusion of multiple cell types in an ECM-like environment to establish 3D epithelial models could potentially help understand and represent realistic epithelial responses following external stimuli.

Here, we described the culture of airway epithelial cells on a tissue-engineered model that mimics the tracheobronchial region and surrounding tissues [13]. Similar epithelial barrier formation for Transwell^®^ and CHyA-B epithelial monocultures was detected, which correlates with previous findings for this scaffold [13]. However, inclusion of co-cultures conditions in Transwell^®^ cultures led to increased TEER values compared to monocultures, which was not seen in CHyA-B cultures as barrier formation remained similar to monoculture of epithelial cells (Figure S1). Reported TEER values from epithelial cultures in CHyA-B match those reported previously for human primary epithelial cells and the ones observed for Calu-3 cells in conventional 2D Transwell^®^ insert models [13,51,52,53]. Reduction of TEER values upon culture on 3D matrices has also been described in airway and intestinal mucosal models and proved to be a more accurate reflection of in vivo barrier formation while inclusion of supporting cell types leads to a faster barrier formation and increased TEER readings [50,54,55]. It has been described that a reduction of TEER upon epithelial culture on 3D environments arises from the formation of less strong tight junctions without affecting barrier formation and do not represent weaker epithelial barrier integrity [56]. Barrier integrity (TEER values) did not fully recover in CHyA-B epithelial monocultures were exposed to bleomycin (Figure 5A). Bleomycin has been shown to impair barrier function and cause severe lung damage and toxicity in animal model as well as in mice-derived lung epithelial cells [57,58,59,60]. Albeit dosing comparison between in vivo and in vitro models can be challenging to interpret, the use of an increased range of 0–1000 mU/mL of bleomycin showed comparable decreased viability in an in vitro model comprising lung epithelial cells as we herein report, which will result in reduced TEER values due to damaged barrier properties [60]. Interestingly, inclusion of Wi38 fibroblasts in CHyA-B cultures led to greater TEER recovery relative to untreated samples (Figure 5B) highlighting the importance of co-culture models to better mimic epithelial responses and epithelial-fibroblasts crosstalk in 3D airway models [28]. The presence of an adjacent fibroblast sublayer has been described to influence barrier formation via growth factor secretion and deposition of ECM components in a human intestinal mucosa model [61]. Therefore, it is expected that epithelial cells cultured in the presence of lung-derived fibroblast will develop enhanced barrier properties towards toxic stimuli as the protective role of fibroblast in airway cells has already been described [62].

Differential toxicological responses were detected between Transwell^®^ and CHyA-B cultures with higher toxicity seen in CHyA-B epithelial co-cultures, demonstrating a greater sensitivity to bleomycin exposure. However, the release of LDH from epithelial monocultures and co-cultures on CHyA-B scaffolds was maintained relative to untreated cultures, in contrast a spike in LDH release was observed in Transwell^®^ cultures post-bleomycin treatment. LDH is released from cells following tissue damage, we therefore hypothesise Transwell^®^ cultures were damaged following bleomycin treatment, but according to metabolic activity and TEER measurements, the epithelial integrity was maintained and therefore LDH levels were higher relative to untreated samples. However, the low LDH release observed from CHyA-B cultures might be explained by the greater disruption and cell damage elicited by bleomycin to these cultures. As evidenced by lower metabolic activity and the inability to recover TEER values in epithelial monocultures, we speculate fewer cells were present in scaffolds compared to Transwell^®^ cultures after exposure (Figure 4 and Figure 5). This behaviour of increased cell sensitivity to toxicants in 3D versus 2D models has been already reported and it is hypothesised that cells grown on 3D environments could offer more sensitive responses when investigating nanoparticle effects on epithelial cells [63,64]. Although it is challenging to draw similarities with actual human data due to its scarcity, it is believed that higher sensitivity of 3D models might arise due to reduced nutrient supply compared to 2D as well as higher basal oxidative stress, which might better represent in vivo conditions [63,65].

Development of 3D models of the airway also has the potential to better represent immune competent models of the airway by combining airway cells with lung and bronchial fibroblasts [66,67,68]. Secretion of cytokines from epithelial cells grown on 3D after ALI conditions are included is linked to the formation of a fully functional epithelium and better represent fibroblast-epithelial interactions [12]. Apical cytokine secretion from 2D cultures was higher in comparison to basolateral secretion as previously reported [69], but this was not seen in 3D cultures. Moreover, increased basolateral secretion of pro-inflammatory cytokines were detected in CHyA-B cultures, in comparison to Transwell^®^ cultures following exposure. In such manner, basolateral evaluation was able to better detect changes in cytokine secretion, highlighting the need to further investigate the role of polarised release of pro-inflammatory mediators when assessing inflammatory responses to inhaled toxicants. Increased cytokine secretion upon culture of epithelial cells in 3D has been demonstrated to better resemble in vivo situations and recruitment of immune cells after epithelial damaged is driven by secretion of cytokines and other mediators [70,71,72,73,74]. Increased detection of cytokine secretion in 3D models is believed to arise from better representation of signalling mechanisms that can remain undetected or underrepresented in 2D models [27,75,76]. Therefore, increased cytokine secretion from CHyA-B scaffolds could better mimic airway cells crosstalk and immune response towards external stimuli which can be explained due to the role of ECM environment regulating cytokine secretion [77]. Inclusion of lung derived fibroblasts led to decreased basolateral levels of IL-8 following LPS stimulation compared to monocultures (Figure 3A,B). This reduced airway inflammatory response upon fibroblast incorporation in a 3D environment correlates with the described protective role of fibroblast in the airways [78]. On the other hand, co-culture conditions lead to higher pro-inflammatory cytokine secretions after bleomycin exposure (Figure 6B,D). We hypothesised that the use of multicellular models could provide better understanding of airway pathology and responses to external stimuli, which could be easily detected by monitoring the secretion of key lung pro-inflammatory cytokines. Furthermore, inclusion of other relevant cell types modulating the immune response after toxicant inhalation should be investigated to better understand cellular responses. Establishing multicellular models on scaffolds resembling ECM and the interaction of cells with their surrounding environment could hold promise to better analyse the inflammation profile following irritant exposure. The development of predictive in vitro models that are able to detect early signs of toxicity and adverse inflammatory cues would allow for improved development of inhaled therapeutics without failure during preclinical assessment due to unidentified side effects [79].

## 5. Conclusions

The use of tissue-engineered 3D models of the airway has the potential to increase the understanding of epithelial responses towards toxicological and immunological stimuli. The combination of ALI conditions in epithelial co-culture in vitro models grown in ECM-like environments hold promise to closely mimic in vivo human physiology and establish reliable tools to study inhaled therapies toxicity and inflammatory profile in an early stage of clinical development.

The goal of this study was to evaluate the feasibility of CHyA-B scaffolds as an improved in vitro model to study toxicological and inflammatory responses from epithelial cultures upon exposure to irritant substances. We established standardised challenge conditions for bacterial and drug-mediated toxicity and inflammation, which will be important in comparing relevant in vitro airway models as they emerge. The toxicological profile and secretion of key mediators in airway inflammation was shown to significantly differ when a mature tracheobronchial epithelium was grown in collagen-based scaffolds compared to standard filter cultures. Future work should aim at the assessment of novel inhaled therapies between the presented airway model and conventional 2D in vitro models. Our results highlight the importance of modelling the ECM-like environment present in airway tissues as well as the need to upgrade current in vitro models with co-cultures in comparison to single cell 2D conventional models of the airway.

## Data Availability

Not applicable.

## Supplementary Materials

The following are available online at https://www.mdpi.com/article/10.3390/biomedicines9060631/s1, Figure S1: Barrier integrity from epithelial monocultures and co-cultures on Transwell^®^ inserts and CHyA-B scaffolds. Figure S2: Optimisation of LPS dose response in Calu-3 epithelial cells. Figure S3: Optimisation of drug-mediated challenge in polarised Calu-3 epithelial cells on Transwell^®^ inserts with 1, 10, and 100 μg/mL of bleomycin for a 24 h exposure time. Figure S4: TEER measurements following LPS exposure. TEER recovery of Calu-3 epithelial monocultures (A) and Calu3 Wi38 epithelial co-cultures (B) on Transwell^®^ inserts and CHyA-B scaffolds following bacterial challenge with 10 μg/mL of *P. aeruginosa* derived LPS for 24 h.

## Abbreviations

CHyA-B	collagen hyaluronic acid bilayered
ALI	air-liquid interface
3D	three-dimensional
2D	two-dimensional
ECM	extra cellular matrix
CHyA	collagen hyaluronic acid
AcOH	acetic acid
DHT	dehydrothermal
DPBS	Dulbecco’s phosphate buffer Solution
EDAC	1-Ethyl-3-(3-Dimethylaminopropyl)carbodiimide
NHS	N-Hydroxysuccinimide
TEER	transepithelial electrical resistance
LPS	lipopolysaccharide
IL-8	interleukin-8
LDH	lactate dehydrogenase
IL-6	interleukin-6
TNF-α	tumour necrosis factor alpha
ELISA	enzyme-linked immunosorbent assay
