# Supplementary material for: A Tissue-Engineered Tracheobronchial In Vitro Co-Culture Model for Determining Epithelial Toxicological and Inflammatory Responses
Source: Biomedicines. 2021 Jun 2;9(6):631. doi: 10.3390/biomedicines9060631 (PMC8226664; doi:10.3390/biomedicines9060631)
Supplement: Supplementary file 1 [file biomedicines-09-00631-s001.zip › biomedicines-1229240-supplementary.pdf]

biomedicines

MDPI

Article

A Tissue-Engineered Tracheobronchial In Vitro Co-Culture Model for Determining Epithelial Toxicological and Inflammatory Responses

Luis Soriano [1,2,3], Tehreem Khalid [1,2,4], Fergal J. O'Brien [2,3,4,5], Cian O'Leary [1,2,3,4] and Sally-Ann Cryan [1,2,3,4,5,*]

Supplementary materials

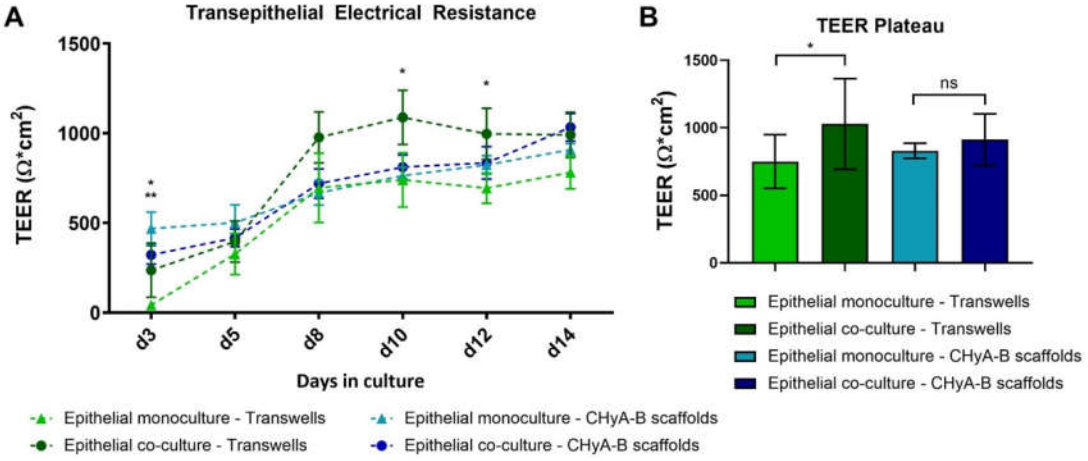

Figure S1: Barrier integrity from epithelial monocultures and co-cultures on Transwell® inserts and CHyA-B scaffolds. (A) TEER values measured at day 3, 5, 8, 10, 12, and 14 from Calu-3 epithelial monocultures (light green) and Calu-3 Wi38 epithelial co-cultures (dark green) on Transwell® inserts; and Calu-3 epithelial monocultures (light blue) and Calu-3 Wi38 epithelial co-cultures on CHyA-B scaffolds under ALI conditions. (B) Average TEER values of epithelial cultures following plateau of electrical resistance measured at day 10, 12, and 14. Results displayed as mean ± SEM ($n = 7$; *$p < 0.05$, ** $p < 0.01$)

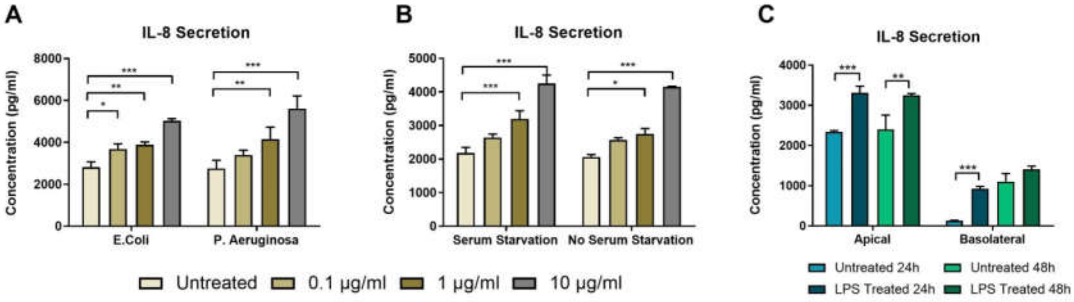

Figure S2. Optimisation of LPS dose response in Calu-3 epithelial cells. (A) IL-8 secretion from non-polarised Calu-3 epithelial cells following LPS exposure with 10, 1, and 0.1 µg/mL of *E. coli* and *P. aeruginosa* derived LPS. (B) IL-8 secretion from non-polarised Calu-3 epithelial cells following LPS exposure 10, 1, and 0.1 µg/mL of *P. aeruginosa* derived LPS in 1% (serum starvation) and 10% (no serum starvation) serum Calu-3 medium. (C) IL-8 secretion from polarised Calu-3 epithelial cells on Transwell® inserts

following 10 µg/mL of *P. aeruginosa* derived LPS for an exposure time of 24 and 48 h. Cytokine release was quantified in both apical and basolateral compartments after bacterial challenge. Results displayed as mean ± SEM (n = 1 performed in quadruplicates; * p < 0.05, ** p < 0.01, *** p < 0.001).

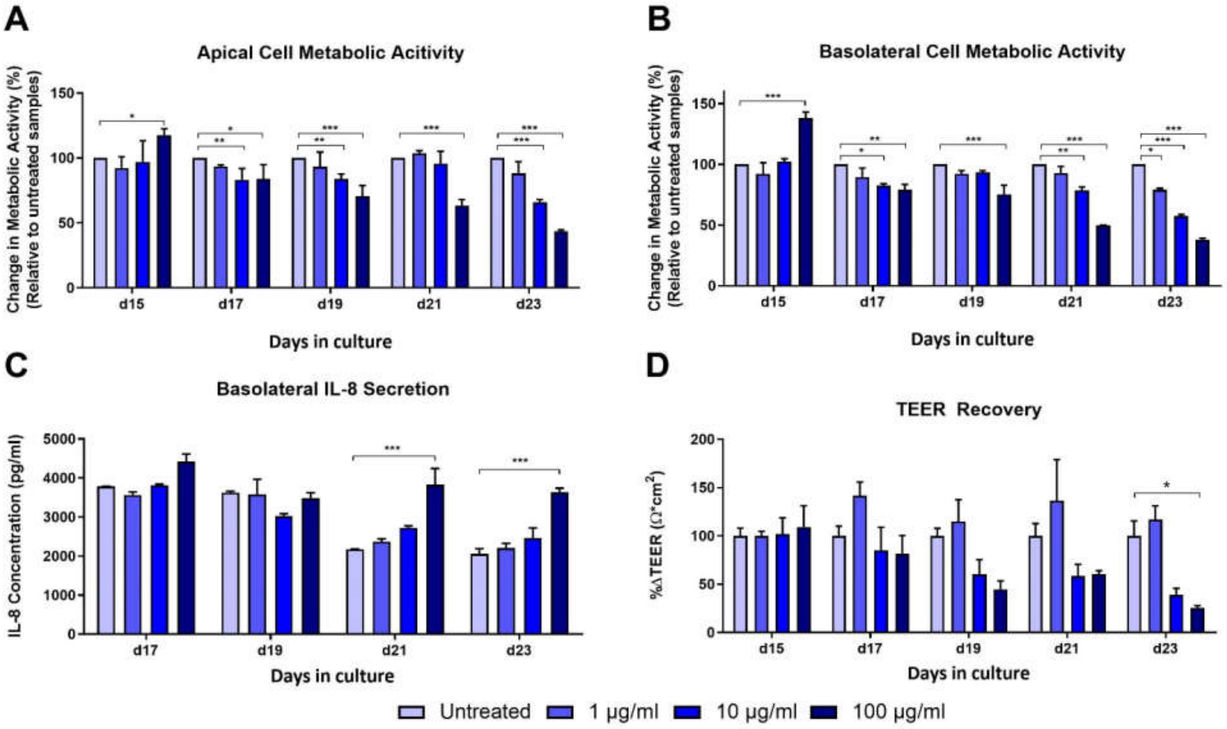

Figure S3. Optimisation of drug-mediated challenge in polarised Calu-3 epithelial cells on Transwell® inserts with 1, 10, and 100 µg/mL of bleomycin for a 24 h exposure time. (**A**,**B**) Apical and basolateral cell metabolic activity from polarised Calu-3 epithelial cells. (**C**) Basolateral IL-8 secretion from polarised Calu-3 epithelial cells. (**D**) TEER recovery from polarised Calu-3 epithelial cells on Transwell® inserts, TEER recovery values normalised to the recovery of untreated samples. Results displayed as mean ± SEM (n = 1 performed in quadruplicates; * p < 0.05, ** p < 0.01, *** p < 0.001).

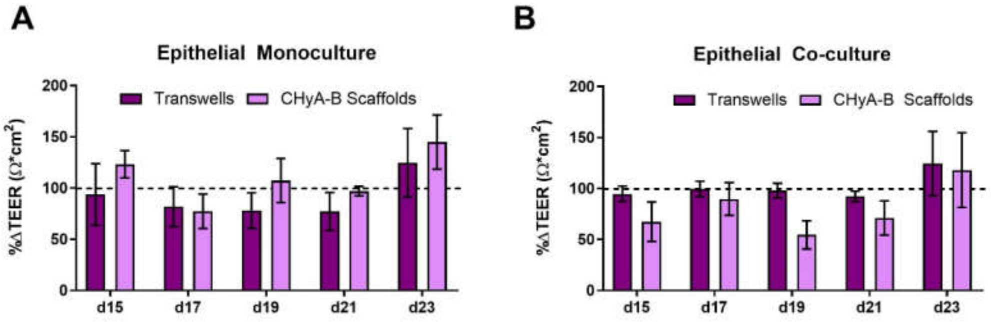

Figure S4: TEER measurements following LPS exposure**.** TEER recovery of Calu-3 epithelial monocultures (**A**) and Calu3 Wi38 epithelial co-cultures (**B**) on Transwell® inserts and CHyA-B scaffolds following bacterial challenge with 10 µg/mL of *P. aeruginosa* derived LPS for 24 h. TEER recovery values normalised to the recovery of untreated cultures. Results displayed as mean ± SEM (n = 3).
